# Improving integrated care: modelling the performance of an online community of practice

**DOI:** 10.5334/ijic.1200

**Published:** 2014-03-10

**Authors:** Ángel Díaz-Chao, Joan Torrent-Sellens, David Lacasta-Tintorer, Francesc Saigí-Rubió

**Affiliations:** Applied Economics Department I, Rey Juan Carlos University, Madrid, Spain; Department of Economic and Business, Open University of Catalonia, Barcelona, Spain; Unitat de Suport a la Recerca Metropolitana Nord, IDIAP Jordi Gol, Santa Coloma de Gramenet, Spain; Department of Health Science, Open University of Catalonia, Barcelona, Spain

**Keywords:** integrated care, community of practice, telemedicine, teleconsulting, partial least squares modelling

## Abstract

**Introduction:**

This article aims to confirm the following core hypothesis: a Community of Practice's use of a Web 2.0 platform for communication between primary and hospital care leads to improved primary care and fewer hospital referrals. This core hypothesis will be corroborated by testing a further five partial hypotheses that complete the main hypothesis being estimated.

**Methods:**

An ad-hoc questionnaire was designed and sent to a sample group of 357 professionals from the Badalona-Sant Adrià de Besòs Primary Care Service in Catalonia, Spain, which includes nine primary care centres and three specialist care centres. The study sample was formed by 159 respondents. The partial least squares methodology was used to estimate the model of the causal relationship and the proposed hypotheses.

**Results:**

It was found that when healthcare staff used social networks and information and communication technologies professionally, and the more contact hours they have with patients, the more a Web 2.0 platform was likely to be used for communication between primary and hospital care professionals. Such use led to improved primary care and fewer hospital referrals according to the opinions of health professionals on its use.

**Conclusions:**

The research suggests that the efficiency of medical practice is explained by the intensity of Web 2.0 platform use for communication between primary and specialist care professionals. Public policies promoting the use of information and communication technologies in communities of practice should go beyond the technological dimension and consider other professional, organisational and social determinants.

## Introduction, comprising background and problem statement

In a context of health spending containment, primary care plays a fundamental role because it is able to potentially offer effective services to prevent unnecessary hospital admissions, to improve the immediacy of care and to reduce waiting lists [1]. However, a characteristic feature of primary care clinics is that they have to attend to a high number of patients suffering from a whole host of health problems of which a considerable amount is clinically and socially complex. This means that physicians and nurses have to deal with various aspects simultaneously, which may raise a multitude of issues in clinical practice that require an effective system to search for information and solve problems [2]. Clinical sessions and individual conversations (in person and over the phone), together with specialist care, are options that allow such issues to be resolved when the complexity requires it [3]. Given that health systems are burdened by an ageing population, in a political context of health expense reduction, communication between primary and specialist care is not easy, quick or effective, and this leads to many referrals to specialist care (hospitalisation or specialist consultations. The result is, more often than not, excessive delays in appointments [1].

Given these communication difficulties, many protocols for referral and communication as well as several telemedicine approaches have been tried out in recent years. Of these, email and videoconferencing have proven to be of particular benefit in terms of efficiency, cost-effectiveness and improved medical care [4]. A number of studies that have assessed healthcare professionals' levels of satisfaction with the use of e-Health solutions have produced good results in terms of improved medical care and use of time [5, 6].

Newer still is the creation of communities of practice in the field of healthcare. Described as a group of people who share an interest in a domain of human endeavour and engage in collective learning that creates bonds among them [7], let healthcare professionals the chance to build knowledge among them working at different levels of care [8]. These virtual communities have proven capable of solving healthcare professionals' information and communication problems in a much simpler way. Moreover, they have been found to improve the functioning of organisations by generating the kind of tacit knowledge that emerges from interactions among colleagues [8, 9].

However, according to the both systematic reviews [10] and [11], there is still little evidence to show whether the use of communities of practice, which rely heavily on social network and information and communication technology use, actually leads to improved efficiency in the integration across primary and hospital care services [12].

Hence, the aim of this article is to study the use of communities of practice to improve current care provision and optimisation of use of healthcare resources. To this end, the results obtained from a Community of Practice's use of a Web 2.0 platform for communication between primary and hospital care will be analysed. This community of practice includes healthcare professionals from primary care centres and specialists from several hospitals in Badalona and Sant Adrià de Besòs (two cities in the Barcelona metropolitan area, Spain). Specifically, we shall investigate the link between the use of this Web 2.0 platform and: (1) improved primary care and (2) fewer hospital referrals according to the opinions of health professionals on its use.

## Theory and methods

### Hypotheses and model

The main aim of this article is to test the following core hypothesis:

Hypothesis 1. A Community of Practice's use of a Web 2.0 platform for communication between primary and hospital care leads to improved primary care and fewer hospital referrals.

This core hypothesis will be corroborated by testing a further set of five partial hypotheses that complete the model of the causal relation being estimated. The model's partial hypotheses are:

Hypothesis 2. When healthcare staff uses social networks for professional purposes in healthcare, the more the Web 2.0 platform is likely to be used.

Hypothesis 3. The more contact hours healthcare professionals have with patients, the more the Web 2.0 platform is used.

Hypothesis 4. The more contact hours healthcare professionals have with patients, the better the outcomes from their activities improved primary care and fewer hospital referrals according to the opinions of health professionals on its use.

Hypothesis 5. The more healthcare staff uses social networks for professional purposes in healthcare, the more information and communication technologies are used in care practice.

Hypothesis 6. The more information and communication technologies are used in care practice, the more contact hours healthcare professionals have with patients.

**Figure 1.**
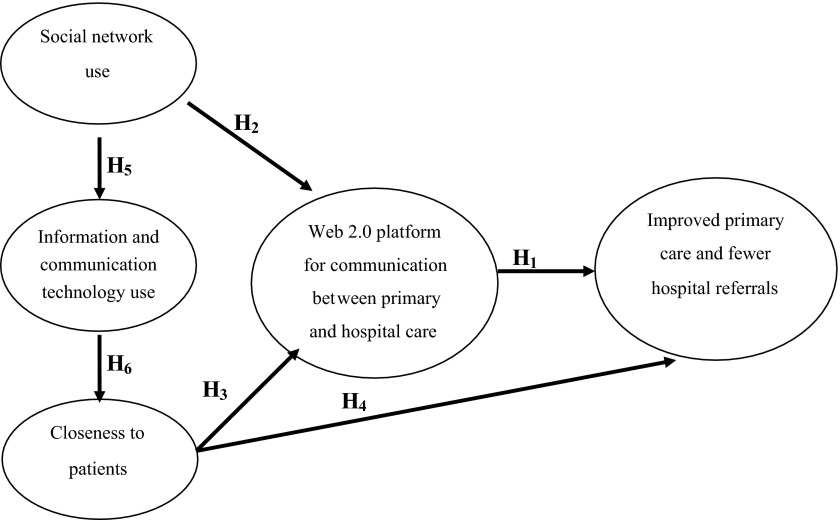
Model and hypothesis to be tested.

Figure 1 shows the model and the explanatory hypotheses of improved primary care and fewer hospital referrals.

## Data collection, empirical methodology and validation

Our paper tries to confirm how using Web 2.0 platform for communication between primary and specialist care leads to improved primary care and fewer hospital referrals. Our empirical approach is based on obtaining and contrasting data provided by the opinions of health professionals. The main advantage of this approach is that this data, of qualitative origin, captures the opinions of the health professionals on the results of the use of this telemedicine approaches as empirical data on improvements to care and resource usage. This methodology allows for the comparison of opinions of professionals in various fields in the use of telemedicine. Finally, the methodology used behind the factors that explain the perceived productivity of information and communication technologies has been widely used in other areas of analysis, e.g., in the analysis of the effect of information and communication technologies on the productivity of the companies or organisations [13, 14].

The research presented in this article is the result of a collaboration between the Badalona-Sant Adrià de Besòs Primary Care Service in Catalonia, Spain and the Open University of Catalonia. The Badalona-Sant Adrià de Besòs primary care service includes nine primary care centres and three specialist care centres: Germans Trias i Pujol University Hospital, Badalona Municipal Hospital and the Barcelonès Nord International Health Unit, with a total of 624 health professionals. These centres serve 227,151 inhabitants.

The digital application being assessed is called ECOPIH (Online Communication Tool between Primary and Hospital Care). It is a community of clinical practice that uses the e-Catalunya platform (http://ecatalunya.gencat.cat) as its content management system. It enables users to share information quickly and easily thanks to its numerous applications, the incremental deployment options and easy integration with well-known environments. A series of sub-groups have been created on the system (one for each active specialty). The following tools are available within each group: (1) a forum where queries about clinical cases are raised for consultation; (2) a document repository and an image repository; (3) a blog where news that people want to share is published; and (4) a calendar and a tool for online document editing [15]. The forum rules of use include respect for the confidentiality of the patient and the identification of the healthcare professional involved. Posts are not moderated prior to publication, though they are reviewed to ensure that they comply with the rules. The platform has a notification system that allows members to receive daily, weekly or monthly emails, containing updates on news available on ECOPIH.

In order to analyse the effect that Web 2.0 platform use (understood as a continually updated service that improves the more people use it and one which consumes and remixes data from multiple sources, including individual users, creating network effects through an "architecture of participation” [16]) has on the outcomes from primary and hospital care practice, an ad-hoc questionnaire was designed. The questionnaire was divided into three sections: (1) sociodemographic and professional background; (2) access to and use of information and communication technologies in professional and personal settings; and (3) perceptions and use of the Web 2.0 platform. Two versions of the questionnaire were produced; one for primary care professionals and another for specialist care professionals. While most of the questions were common to both, some differed depending on the area of work. The questionnaires were anonymous and optional, and potential respondents were informed about the scientific objectives pursued and the confidentiality of data obtained.

An invitation to complete the questionnaire was sent to 357 primary care professionals (physicians and nurses) bounded by common practice in caring for the population of Badalona-Sant Adrià de Besòs primary care service and who were regular users of Ecopih. Among the 357 were 66 supply staff and resident physicians, and to 89 specialist hospital care professionals, who could both freely send the questionnaire to other colleagues. The questionnaire was completed by a total of 169 professionals, who formed the study sample and determined the overall response rate, which was slightly lower than half of the sampling universe. The fieldwork was carried out between 1 December 2011 and 31 January 2012.

Of the respondents, 82.8% (*n* = 140) was primary care professionals and 17.2% (*n* = 29) was hospital care professionals. Of those respondents, 64.5% (*n* = 109) was physicians, 33.7% (*n* = 57) was nurses and the rest (1.8% (*n* = 3) were physiotherapists, psychologists and social workers. The mean age of the respondents was 46.9 years, and women accounted for 72.2% (*n* = 122) of the respondents.

The constructs and variables described below were used to test the research hypotheses. The *outcomes from healthcare professionals' activities* construct was captured by means of two variables reflecting the respondents' opinions about whether the Web 2.0 platform led to improved primary care and fewer hospital referrals. Both variables are discrete and reflect the respondents' opinions on a scale from 1 (totally disagree) to 5 (totally agree). The *Web 2.0 platform use* construct was captured by means of a categorical variable reflecting frequency of use (0 = never; 1 = occasionally; 2 = monthly; 3 = weekly; 4 = daily) of the Web 2.0 platform to read published content, to raise queries with specialists, to post something (a blog entry, comments, references, etc.), to take part in discussions or to share documentation.

The *social network use* construct was captured by means of a binary variable (0 = not used; 1 = used) reflecting whether healthcare professionals habitually used one of the following social networks in their healthcare practice: Facebook, Twitter, Google+, LinkedIn, a personal blog or someone else's blog. The *information and communication technology use* construct was captured by means of a binary variable (0 = not used; 1 = used) reflecting whether healthcare professionals habitually used one of the following digital devices in their healthcare practice: a smartphone, a desktop computer, a laptop, a tablet, a PDA, an iPad or other similar device. Finally, the *closeness to patients* construct, which for the purposes of this paper refers to the amount of contact hours with patients, was measured by means of a variable that, according to professional categories, approximated healthcare professionals' frequency of contact with patients using the Web 2.0 platform. This categorical variable has four values: 0 = psychologist, physiotherapist, occupational therapist, etc.; 1 = nurse; 2 = specialist physician; 3 = primary care physician (Table 1).

The partial least squares algorithm was used to estimate the model and the proposed hypotheses [17, 18]. This technique was chosen over others, such as structural equation modelling, for several reasons. First, it is a highly evolved validated prediction modelling technique in which data multinormality can be relatively relaxed [19, 20]. Second, it allows causal relationships between latent dimensions and measurement variables to be determined. Third, in addition to estimating causal relationships between latent variables, the partial least squares methodology allows formative latent variables to be calculated by setting the weights of each explanatory (and observable) variable. Estimating models of this type naturally requires appropriate measurements of goodness-of-fit and robustness of analysis to validate the proposed model.

We shall therefore present the validation of the proposed structural and measurement models before proceeding to report on the results. Regarding the assessment of the measurement model, several tests were conducted while taking account of the fact that the latent variables included in the model were formative constructs. Thus, on the one hand, the analysis of content validity allowed us to check whether the indicators had captured the full scope of the model [21]. The analysis confirmed that the indicators had been appropriately selected [22].

On the other hand, the analysis of construct reliability allowed us to check the internal consistency of the measurement model. The validity and reliability of each indicator and construct were assessed and no multicollinearity problems were found. The variance inflation factor was lower than the threshold value of 3.3 [23]. All of the indicators were significant (*p* < 0.10) as detailed below in the section on the results of estimation using partial least squares (Table 2).

Two further validation measurements were made: construct validity and discriminant validity. For construct validity, the correlations between the constructs were lower than 0.5. Pairwise, they were therefore sufficiently different. For discriminant validity, Table 3 shows the cross-loadings obtained from the correlations between each item and each latent variable. The coefficient between each indicator and respective latent was high in all cases.

After analysing the measurement model validation criteria, we dealt with the structural model validation criteria (Table 4). There were two criteria: model validity and predictive power. For model validity, the estimation was performed using 200 bootstrap resamples. Thus, the *R* ^2^ result obtained to test Hypothesis 1 (improved primary care and fewer hospital referrals variable) was 0.219. While the result was relatively moderate, it did not undermine the validity of the estimations as a whole, because the causal effects obtained were in accordance with expectations. Moreover, all the variables showed the expected signs, and *t* statistics were sufficiently high. Indeed, the model's predictive power was robust because the coefficients obtained for the *Q* ^2^-statistic communality values were greater than zero for the improved primary care and fewer hospital referrals variable (*Q* ^2^ = 0.564 and *Q* ^2^ = 0.527, respectively). The *Q* ^2^-statistic cross-validated redundancy values were also greater than zero (0.154 and 0.156, respectively), thus supporting the predictive relevance of the whole model. (The *Q* ^2^-statistic was evaluated by applying the blindfolding procedure with an omission distance of 7. The proposed threshold value is *Q* ^2^ > 0; a higher *Q* ^2^ value means a higher predictive relevance of the model).

## Results

Partial least squares estimation of the described model accepted and confirmed the proposed hypotheses (Tables 5 and 6). First, a Community of Practice's use of a Web 2.0 platform for communication between primary and hospital care led to improved care practice (*β* = 0.415, *p* < 0.001, Hypothesis 1). In this respect, the results construct were evidenced by improved primary care (*β* = 0.474, *p* < 0.10) and fewer hospital referrals (*β* = 0.581, *p* < 0.05).

Likewise, closeness to patients had a direct positive effect on the results for Web 2.0 platform use (*β* = 0.148, *p* < 0.10, Hypothesis 4). In other words, the higher the frequency of contact with patients, the better the results for Web 2.0 platform use. Similarly, closeness to patients had indirect effects on the results for care practice through Web 2.0 platform use. Its effect on Web 2.0 platform use was significant and positive (*β* = 0.147, *p* < 0.05, Hypothesis 3). The total effect of the closeness to patients' variable on the results construct was 0.209.

Furthermore, professional information and communication technology use positively and significantly explained closeness to patients (*β* = 0.138, *p* < 0.1, Hypothesis 6). The indirect effect of information and communication technology use on the results for Web 2.0 platform use was produced by the combination of closeness to patients and Web 2.0 platform use (0.029). However, this effect was not statistically significant at standard levels (*t* = 1.242).

Finally, professional social network use explained professional information and communication technology use (*β* = 0.487, *p* < 0.001, Hypothesis 5) and Web 2.0 platform use (*β* = 0.357, *p* < 0.05, Hypothesis 2). These effects were greater for women than for men. Thus, the total effect of professional social network use on improved primary care and fewer hospital referrals was 0.162 (*p* < 0.05).

**Figure 2.**
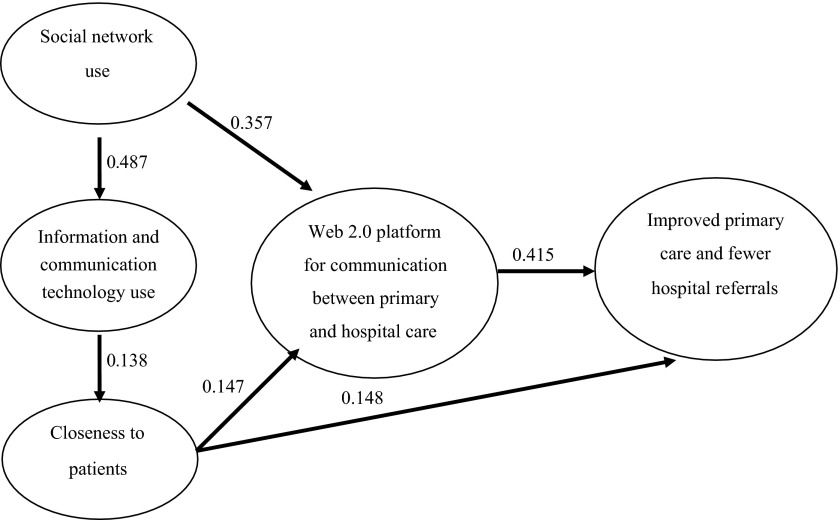
Outer weights diagram (mean).

To sum up, when healthcare staff used social networks and information and communication technologies professionally, and the closer they were to patients, the more a Web 2.0 platform was likely to be used for communication between primary and hospital care professionals. Such use led to improved primary care and fewer hospital referrals (Table 3; Figure 2).

## Discussion

In this article, we postulated that the use of a Web 2.0 platform for communication between primary and hospital care could improve the performance of care practice. Using a partial least squares methodology to determine causal relationships, and a study sample of 169 healthcare professionals who were users of a Web 2.0 platform, we established that professional social network and information and communication technology use, and amount of contact hours with patients, explained the intensity of Web 2.0 platform use. A response rate of 47.3% to the questionnaire is considered positive in non-mandatory online surveys. In addition, the intensity of Web 2.0 platform use improved care practice, leading to improved primary care and fewer hospital referrals.

Our working hypothesis was based on evidence available in the literature. It has been suggested that the development of Web 2.0 platforms for communication between care levels enables issues arising in primary care professional practice to be resolved [24]. In this respect, a meta-analysis performed on interactive communication between primary and hospital care physicians in the areas of psychiatry and endocrinology found that this type of communication increased collaboration effectiveness and was associated with important clinical advances in general, and improved patient outcomes in particular [25].

The research presented here provides new evidence because it found that according to the opinions of health professionals on its use, Web 2.0 platform use could lead to improved primary care and fewer hospital referrals. This finding is very much in keeping with some partial findings already described in the literature, particularly in the field of teleconsultation. In a study conducted on joint care practice between primary care physicians and general medicine specialists, who saw patients that were due be referred to hospital, the referral rate fell by 22% and hospital care effectiveness improved [26]. Other studies have shown that a second opinion was very useful in reducing the need to visit a specialist for an ECG [27]. Previous studies showed that, through teleconsultation, physicians managed to avoid up to 95% of cardiology consultations in patients with a high cardiovascular risk [28]. The effectiveness of teleconsultation has also been demonstrated in other specialities [29–31]. Studies in the area of asynchronous teledermatology (image sending and deferred viewing) concluded that it was possible to reduce the number of referrals by up to 50%, and even 69% [32]. In a study for Finland, teleconsultation with a hospital via the internet allowed 52% of patients to continue to be treated in the primary care setting, thus avoiding their referral to hospital. Also in Finland, it has already been concluded that teleconsultation between primary and hospital care improves clinical effectiveness, reduces direct costs, increases productivity and is cost-effective [33].

While there are many medical consultation methods that make use of information and communication technologies, evidence in the areas of social networks and communities of practice in integrated care is rather scarce. Most published studies have shown that teleconsultation and data transfer are limited to primary care physicians and specialists, without allowing other professionals to get involved. In this context, the creation of communities of clinical practice has proven to be a useful means of solving problems and improving the functioning of healthcare organisations [11, 12].

Notwithstanding the above, a reduction in referrals has many other explanatory factors that were not contemplated in our study, such as access to supplementary tests or the healthcare professionals' motivation for using Web 2.0 platforms [34]. The Web 2.0 platform for communication between primary and specialist care professionals contemplated in our study does not permit direct access to supplementary tests. The healthcare professionals' motivation therefore appears to be the key factor in the success of the platform. In this context, and given their good results, there are studies suggesting that there should be incentives to get healthcare professionals to use such platforms [6, 9].

## Conclusion

In the context of communities of practice, our research concludes that the intensity of Web 2.0 platform use for communication between primary and specialist care professionals in the integration across primary and hospital care services is determined by technological, organisational and social factors. This could suggest that the position that professionals have within the healthcare structure, and particularly their closeness to patients and their professional experience of using social networks and information and communication technologies is crucial in explaining the use of such platforms. Therefore, public policies promoting the use of information and communication technologies in the field of medicine in general, and in communities of practice in particular, should go beyond the purely technological dimension and consider other professional and social determinants, as well as those of a contextual and organisational nature.

In this respect, the research presented here has a number of limitations, particularly sample size, the constructs and indicators used, and the lack of a longer time series. Nevertheless, given the importance of Web 2.0 platforms of this type to the performance of the healthcare system, and the availability of data for a group of professionals and medical practices, it brings new evidence – albeit preliminary – to debate. Recommendations for future study therefore include increasing sample size, extending the analysis period, incorporating new explanatory dimensions (particularly in relation to organisational, social, cultural and human resources aspects), and performing a more detailed analysis of efficiency and productivity indicators and determinants of medical practice.

While the results obtained establish causal relationships in the explanation of hospital referrals and the use of the community-of-practice tool, the nature of the data obtained suggests the need for future study. First, it would be interesting to compare impact, in probabilistic terms, by using discrete choice models based on healthcare professionals' perception data. And second, this model should be supplemented with quantitative data on the effective number of hospital referrals by means of a cost-benefit analysis.

## Authors' contributions

The survey was principally undertaken by Fancesc Saigí Rubió and David Lacasta, supported by the statistical modelling, contributed by Ángel Díaz-Chao and Joan Torrent. Fancesc Saigí Rubió is the guarantor of the article.

## Figures and Tables

**Table 1.**
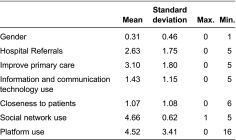
Descriptive statistics

**Table 2.**
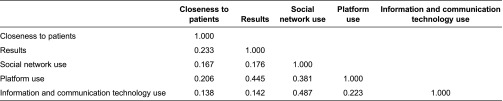
Latent variable correlations

Source: Own elaboration.

**Table 3.**
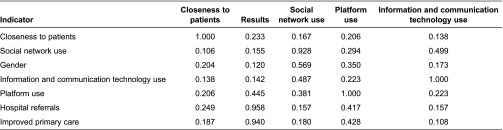
Cross-loadings

Source: Own elaboration.

**Table 4.**
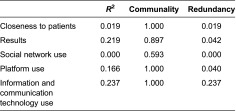
Quality model measurements overview

Source: Own elaboration.

**Table 5.**
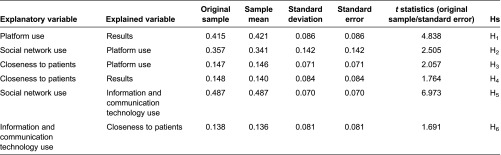
Outer weights of mean, standard deviation and *t* statistics

Hs: Hypotheses

Source: Own elaboration.

**Table 6.**
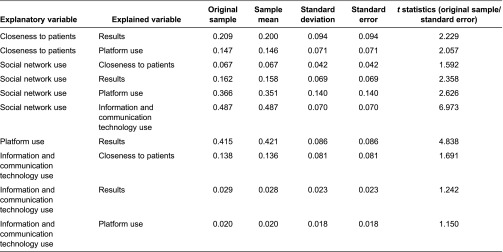
Total effects of mean, standard deviation and *t* statistics

Source: Own elaboration.
